# The *BMC Medicine* breast cancer collection: an illustration of contemporary research and clinical care

**DOI:** 10.1186/s12916-015-0474-5

**Published:** 2015-09-23

**Authors:** Debu Tripathy

**Affiliations:** The University of Texas MD Anderson Cancer Center, 1515 Holcombe, Unit 1354, Houston, 77030 TX USA

**Keywords:** Breast cancer, Clinical trials, Molecular, Targeted therapy, Translational

## Abstract

The field of breast cancer had witnessed clear improvements in survival and less morbidity over the last few decades owing to earlier detection as a result of public awareness and screening, as well as treatments involving the disciplines of surgical, radiation and medical oncology along with advances in imaging and pathological diagnostics. However, in the last 5–10 years, newer assays and biological therapies have begun to cross new boundaries with higher rates of cure seen in more aggressive cancers. Even though metastatic breast cancer remains incurable, some, but not all, subsets of patients with breast cancer are living longer and more productive lives. Many challenges still remain, and the development of team science coupled with collaborative clinical research and care is expected to accelerate advances along this trajectory.

## Introduction

The one-year anniversary of our *Spotlight on breast cancer* collection is a timely opportunity to review the snapshot of accomplishments in research and new care standards represented in this series of original articles and reviews. Global interest in breast cancer owing to its commonality and its growing incidence has created a surge in research funding and scientific output that is now being translated into clinical gains. Relative mortality and morbidity have clearly been favorably impacted, but recurrence and incurability of advanced disease remain problematic. Specific drivers of cancer initiation, progression, metastasis, and drug resistance are elusive, and their interactions with normal biological pathways are complex and highly individualized to both tumor biology and host factors. The convergence of data-dense analytical technologies in genomics, transcriptomics, epigenetics, and proteomics, with linkage to well-annotated human tissue sets and prospective clinical trials, have begun to yield new information and insights. In our breast cancer collection, we attempt to provide examples that stitch together the larger picture of where we have been and where we are going. The goalpost should be crystal clear—better clinical outcomes, in terms of both survival and quality of life.

## The connection of estrogen and breast cancer: from prevention to treatment

In breast cancer, hormone receptor pathways, initially targeted as far back as 1896 with the first report of a clinical response to oophorectomy, remain a key focus of newer discoveries [1]. This is expected, because cancers are a distillation of continuous selection of the fittest from cumulative genetic and epigenetic insults, and the normal cellular “toolkit” is the most likely to be co-opted in this process. In the case of breast cells, estrogen is a key developmental and growth stimulus—the cell is essentially programmed to respond to estrogen. Assuming the stepwise (or parallel) model of serial random (or possibly biased) alterations leading to cancer, it would stand to reason that many of these events will involve the estrogen receptor signaling apparatus. Moreover, these phases would range from early pre-neoplasia to late events such as invasion and metastasis.

Might suppression of estrogen pathways therefore yield a preventive effect, just like statins and antihypertensive agents are postulated to lower the risk of coronary disease? Oophorectomy is clearly associated with a lower risk of breast cancer, albeit at a cost of symptoms and possible longer-term effects of estrogen deficiency, such as osteoporosis [2]. In our breast cancer series, Vogel summarizes the long history of the development of selective estrogen receptor modulators (SERMs) as breast cancer treatment and, ultimately, prevention [3]. In the case of invasive breast cancer, SERMs and aromatase inhibitors, through different mechanisms, reduce recurrence of breast cancer by one half and mortality by one third. In the preventive setting, however, the pivotal trials could not practically be powered for survival impacts. Yet this raises the key question as to whether these preventive therapies are simply delaying the clinical onset of breast cancer or actually preventing it. It may not make a difference to the individual whose cancer is delayed as to never surface clinically in their lifetime. However, hormonal suppression may simply shift selective pressures in other directions—as suggested by recent preliminary findings of non-significantly higher estrogen receptor-negative breast cancer rates in the IBIS-I tamoxifen prevention trial [4]. Still, a 30–50 % reduction in breast cancer with 5 years of preventive therapy, consistent across multiple trials, is a big public health triumph given the morbidity associated with treatments. But even though tamoxifen and raloxifene are approved and recommended for breast cancer prevention for those at even modestly elevated risk, they are remarkably underutilized [5]. Ongoing refinements of SERMs designed to impact other estrogen-associated outcomes, primarily bone and cardiovascular, are a priority, yet challenging given the size and follow times required of the trials. Development of more sophisticated risk models and validated surrogate biomarkers may allow for more efficiently designed trials in the future.

There is overwhelming evidence that adjuvant hormonal therapy with tamoxifen and aromatase inhibitors have improved mortality from hormone receptor-positive early stage breast cancer [6, 7]. Predictors of response other than quantitative measures of estrogen receptor expression [8], however, remain obscure. Artigalás et al. [9] present a systematic review and meta-analysis of inherited polymorphisms of *CYP19A1*, the gene encoding aromatase. This enzyme is present in peripheral tissue—particularly in adipose tissue—and converts androgens to estrogens as the primary source of these hormones in postmenopausal women (and men). Serum estrogen levels are therefore related to body mass index, a known risk factor for breast cancer and breast cancer recurrence—even possibly in male breast cancer as suggested by Humphries et al. [10]. Hence, it is postulated that certain inherited functional variants of *CYP19A1* are related to estrogen levels, risk of breast and uterine cancer, and outcomes in hormone receptor-positive breast cancer [11–13]. This overview of 12 studies, which included early, neoadjuvant, and advanced stage cohorts, showed that the rs4646 single nucleotide polymorphism (SNP) was associated with approximately a doubling time to disease progression, while associations seen with other SNPs in individual studies did not emerge as significant [9]. This study by Artigalás et al. illustrates the need to assess large numbers of studies to make genomic–phenotype associations from broad-scale searches. Their study was limited in size and also by the fact that many SNPs were only reported in one or few of the studies. In addition, associations from data mining exercises need to be confirmed functionally in cell-based or animal models, with the acknowledgement that these are also not perfect and could in fact miss important drivers of physiological behavior. Then, further supportive validation needs to be made, preferably from prospective controlled trials.

In the metastatic setting, tamoxifen and aromatase inhibitors, along with the estrogen receptor down-regulator fulvestrant are all effective in transiently halting progression of disease, although resistance ultimately develops with a wide range of median times to disease progression among patients. Migliaccio et al. have reviewed the evolution of hormonal therapies and more recent developments in combinations of different hormonal therapies [14]. Several pathways have been shown to interact with those mediated by the estrogen receptor, primarily growth-factor pathways as well as the central control of the cell cycle, which are also intimately tied to growth and metabolic pathways as well as multiple other inputs. The last few years have witnessed the development of several drugs that modulate these pathways and augment the clinical benefit of hormonal blockade. Yamamoto-Ibusuki et al. have summarized the background and recent trial results, showing doubling of progression-free survival but, interestingly, no impact on overall survival from the addition of the mTOR inhibitor everolimus to aromatase inhibitor therapy [15–17]. More recently, the cyclin-dependent kinase 4/6 inhibitor palbociclib has been shown to produce even larger augmentations of progression-free survival—both in the first-line setting when added to letrozole [18] and in the second-line setting when added to fulvestrant [19], but, again, without survival benefits at this point in follow-up, possibly due to the limited number of deaths seen so far. Both everolimus and palbociclib are in or entering trials in the adjuvant setting—these finding are eagerly awaited to address the critical questions as to whether metastases and death from breast cancer can be lowered with acceptable toxicities over and above the remarkable gains already achieved with hormonal therapy alone. As Yamamoto-Ibusuki et al. point out, much more work is needed to decipher other components of the PI3K/Akt/mTOR pathway as newer drugs directly inhibit PI3K, or specifically the p110α catalytic subunit that commonly harbors activating mutations, as well as Akt enter clinical testing.

The theme of body mass and energy balance ties in not only to circulating estrogens, but also metabolic pathways involving AMP-activated protein kinase and mTOR as well as metabolic pathways mediated by insulin and insulin like-growth factors. All of these networks have been implicated in cancer development and progression, with recurrent mutations in these pathways seen in diverse cancer types. Many organizations are now recommending a “heart-healthy” lifestyle to lower the risk of cancer and recurrence for many tumor types [20], but optimal exercise and diet regimens remain undefined, and controlled prospective trials with recurrence and mortality endpoints are lacking. However, there are other potential benefits of exercise including management fatigue and other quality of life indices, particularly in patients receiving chemotherapy, There has been interest in developing and formally testing specific exercise regimens that vary based on intensity, duration, and other factors [21]. Travier et al. reported on an a usual care-controlled 18-week supervised program of aerobic and strength exercise with cognitive behavioral support during chemotherapy for early stage breast cancer and found no effect on the primary endpoint of fatigue, but did show less of an increase in fatigue with chemotherapy [22]. Such studies are critical to define optimal approaches and methods to individualize physical activity regimens. Also, long-term sustainability of lifestyle interventions must be achieved for any long-term impact on quality of life and cancer control endpoints.

## Deciphering growth factor pathways and associated networks

In addition to estrogen pathways, other known key breast carcinogenesis drivers include growth factor receptor pathways (e.g., HER2) as well as DNA repair deficiency, which is both an acquired as well as an inherited genotype and phenotype. Targeting the HER2 receptor in the 20 % of cancers in which HER2 is amplified and overexpressed has also improved outcome in the adjuvant setting, comparable to the improvement seen with hormonal therapy, with the use of 1 year of the humanized anti-HER2 antibody trastuzumab [23]. Predictors of benefit and markers of resistance have been described [24], but not widely confirmed and validated; hence, none are used in the clinic to help guide therapy (other than the determination of HER2 status itself). The use of multiparametric analysis at the genomic, gene expression, and proteomic levels, along with bioinformatics analysis, can discern patterns and identify biological pathways that may not only predict responsiveness, but point to therapeutic targets that could be addressed pharmacologically to augment activity and reverse clinical resistance. This requires that the samples analyzed are sufficient in number and, ideally, derived from a clinical trial where patients were randomized to receive or not receive the therapy in question. Sonnenblick et al. described an original finding after interrogating tissue samples from a prospective randomized trial (FinHER), an institutional cohort, and The Cancer Genome Atlas (TCGA), using gene expression and reverse phase protein array analysis [25]. Their computational plan focused on developing a signal transducer and activator of transcription 3 protein (STAT3) signature that was derived from the institutional dataset and validated on TCGA data, and then tested for its ability to predict distant recurrence on both the institutional and FinHER datasets. The results showed that activated (phosphorylated) STAT3 did not predict outcome, but the signature did—a theme that is repeating in the field of prognostic and predictive biomarkers, specifically, the notion that a pathway signature rather than any specific component might be a better way to link biology to outcome. In fact, this is being reflected in newer classifications of breast cancer that are based on gene expression and other signatures. Determining whether these will ultimately replace conventional single biomarkers need await more studies, most importantly those that are linked to prospective trials that are increasingly being developed with parallel multiparametric correlative studies.

The *Spotlight on breast cancer* collection illustrates trends in breast cancer basic, translational, and clinical sciences and highlights the blurring lines and interactions between laboratory and clinical investigation that will be necessary to move the field forward. Continued technological advances coupled with a commitment to making every clinical trial not only generate clinical outcomes but also biological readouts that can be integrated to fashion newer strategies will be essential in our goal to help patients with breast cancer live longer and fuller lives.

## References

[1] Beatson GT (1896). On the treatment of inoperable cases of carcinoma of the mamma: suggestions for a new method of treatment, with illustrative cases. Lancet.

[2] Rocca WA, Grossardt BR, de Andrade M, Malkasian GD, Melton 3rd LJ. Survival patterns after oophorectomy in premenopausal women: a population-based cohort study. Lancet Oncol. 2006;7:821–8. 2006. 10.1016/S1470-2045(06)70869-5 17012044

[3] Vogel VG (2015). Ongoing data from the breast cancer prevention trials: opportunity for breast cancer risk reduction. BMC Med.

[4] Cuzick J, Sestak I, Cawthorn S, Hamed H, Holli K, Howell A (2015). Tamoxifen for prevention of breast cancer: extended longterm follow-up of the IBIS-I breast cancer prevention trial. Lancet Oncol.

[5] Waters EA, McNeel TS, Stevens WM, Freedman AN (2012). Use of tamoxifen and raloxifene for breast cancer chemoprevention in 2010. Breast Cancer Res Treat.

[6] Early Breast Cancer Trialists’ Collaborative Group. Aromatase inhibitors versus tamoxifen in early breast cancer: patient-level meta-analysis of the randomised trials. Lancet 2015; published online 23 July. http://www.ncbi.nlm.nih.gov/pubmed/?term=Aromatase+inhibitors+versus+tamoxifen+in+early+breast+cancer%3A+patient-level+metaanalysis+of+the+randomised+trials. (15)61074-1. 10.1016/S0140-6736(15)61074-1 26211827

[7] Early Breast Cancer Trialists Collaborative Group (EBCTCG). Relevance of breast cancer hormone receptors and other factors to the efficacy of adjuvant tamoxifen: patient-level meta-analysis of randomised trials. Lancet. 2011;378:771–84. 10.1016/S0140-6736(11)60993-8 PMC3163848 21802721

[8] Kim C, Tang G, Pogue-Geile KL, Costantino JP, Baehner FL, Baker J (2011). Estrogen receptor (ESR1) mRNA expression and benefit from tamoxifen in the treatment and prevention of estrogen receptor-positive breast cancer. J Clin Oncol.

[9] Artigalás O, Vanni T, Hutz MH, Ashton-Prolla P, Schwartz IV (2015). Influence of CYP19A1 polymorphisms on the treatment of breast cancer with aromatase inhibitors: a systematic review and meta-analysis. BMC Med.

[10] Humphries MP, Jordan VC, Speirs V (2015). Obesity and male breast cancer: provocative parallels?. BMC Med.

[11] Dunning AM, Dowsett M, Healey CS, Tee L, Luben RN, Folkerd E (2004). Polymorphisms associated with circulating sex hormone levels in postmenopausal women. J Natl Cancer Inst.

[12] Low YL, Li Y, Humphreys K, Thalamuthu A, Yi L, Darabi H (2010). Multi-variant pathway association analysis reveals the importance of genetic determinants of estrogen metabolism in breast and endometrial cancer susceptibility. PLoS Genet.

[13] Wang L, Ellsworth KA, Moon I, Pelleymounter LL, Eckloff BW, Martin YN (2010). Functional genetic polymorphisms in the aromatase gene CYP19 vary the response of breast cancer patients to neoadjuvant therapy with aromatase inhibitors. Cancer Res.

[14] Migliaccio I, Malorni L, Hart CD, Guarducci C, Di Leo A (2015). Endocrine therapy considerations in postmenopausal patients with hormone receptor positive, human epidermal growth factor receptor type 2 negative advanced breast cancers. BMC Med.

[15] Yamamoto-Ibusuki M, Arnedos M, André F (2015). Targeted therapies for ER+/HER2- metastatic breast cancer. BMC Med.

[16] Baselga J, Campone M, Piccart M, Burris HA, Rugo HS, Sahmoud T (2012). Everolimus in postmenopausal hormone receptor-positive advanced breast cancer. N Engl J Med.

[17] Piccart M, Hortobagyi GN, Campone M, Pritchard KI, Lebrun F, Ito Y (2014). Everolimus plus exemestane for hormone receptor-positive, human epidermal growth factor receptor-2-negative advanced breast cancer: overall survival results from BOLERO-2. Ann Oncol.

[18] Finn RS, Crown JP, Lang I, Boer K, Bondarenko IM, Kulyk SO (2015). The cyclin-dependent kinase 4/6 inhibitor palbociclib in combination with letrozole versus letrozole alone as first-line treatment of oestrogen receptor-positive, HER2-negative, advanced breast cancer (PALOMA-1/TRIO-18): a randomised phase 2 study. Lancet Oncol.

[19] Turner NC, Ro J, Andre F, Loi S, Verma S, Iwata H (2015). Palbociclib in hormone-receptor–positive advanced breast cancer. New Engl J Med.

[20] Ligibel JA, Alfano CM, Courneya KS, Demark-Wahnefried W, Burger RA, Chlebowski RT (2014). American Society of Clinical Oncology position statement on obesity and cancer. J Clin Oncol.

[21] van Waart H, Stuiver MM, van Harten WH, Geleijn E, Kieffer JM, Buffart LM (2015). Effect of low-intensity physical activity and moderate- to high-intensity physical exercise during adjuvant chemotherapy on physical fitness, fatigue, and chemotherapy completion rates: results of the PACES randomized clinical trial. J Clin Oncol.

[22] Travier N, Velthuis MJ, Steins Bisschop CN, van den Buijs B, Monninkhof EM, Backx F (2015). Effects of an 18-week exercise programme started early during breast cancer treatment: a randomised controlled trial. BMC Med.

[23] Kim M, Agarwal S, Tripathy D (2014). Updates on the treatment of human epidermal growth factor receptor type 2-positive breast cancer. Curr Opin Obstet Gynecol.

[24] Rexer BN, Arteaga CL (2012). Intrinsic and acquired resistance to HER2-targeted therapies in HER2 gene-amplified breast cancer: mechanisms and clinical implications. Crit Rev Oncog.

[25] Sonnenblick A, Brohée S, Fumagalli D, Vincent D, Venet D, Ignatiadis M (2015). Constitutive phosphorylated STAT3-associated gene signature is predictive for trastuzumab resistance in primary HER2-positive breast cancer. BMC Med.

